# Author Correction: A selective inhibitor of mitofusin 1-βIIPKC association improves heart failure outcome in rats

**DOI:** 10.1038/s41467-024-47288-3

**Published:** 2024-04-03

**Authors:** Julio C. B. Ferreira, Juliane C. Campos, Nir Qvit, Xin Qi, Luiz H. M. Bozi, Luiz R. G. Bechara, Vanessa M. Lima, Bruno B. Queliconi, Marie-Helene Disatnik, Paulo M. M. Dourado, Alicia J. Kowaltowski, Daria Mochly-Rosen

**Affiliations:** 1 Department of Anatomy, Institute of Biomedical Sciences, University of Sao Paulo, Sao Paulo, 05508-000 SP Brazil; 2 Department of Chemical and Systems Biology, Stanford University School of Medicine, Stanford, 94305-5174 CA USA; 3 Department of Physiology & Biophysics, Case Western Reserve University, Cleveland, 44106 OH USA; 4 Departamento de Bioquímica, Instituto de Química, Universidade de Sao Paulo, Sao Paulo, 05508-000 SP Brazil; 5 Heart Institute, University of Sao Paulo, Sao Paulo, 05403-010 SP Brazil

Correction to: *Nature Communications* 10.1038/s41467-018-08276-6, published online 18 January 2019

The original version of this Article contained errors in Fig. 1i, Fig. 3e, Supplementary Fig. 6, Supplementary Fig. 8, and Supplementary Fig. 1.

The Tom20 western blot band in Fig. 1i was erroneously presented upside down.

The correct version of Fig. 1 is:
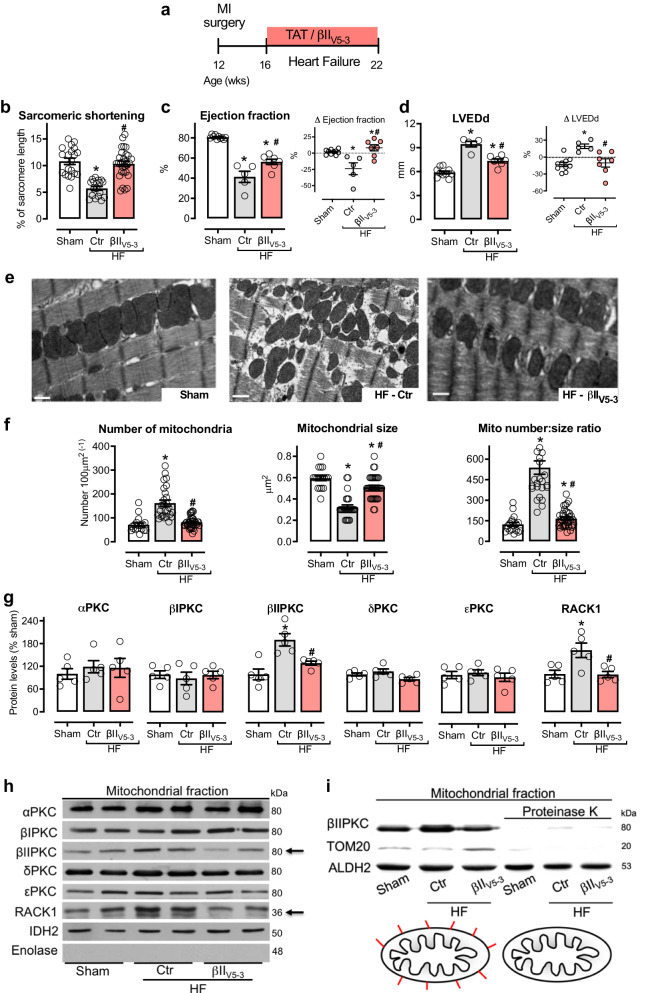


which replaces the previous incorrect version:
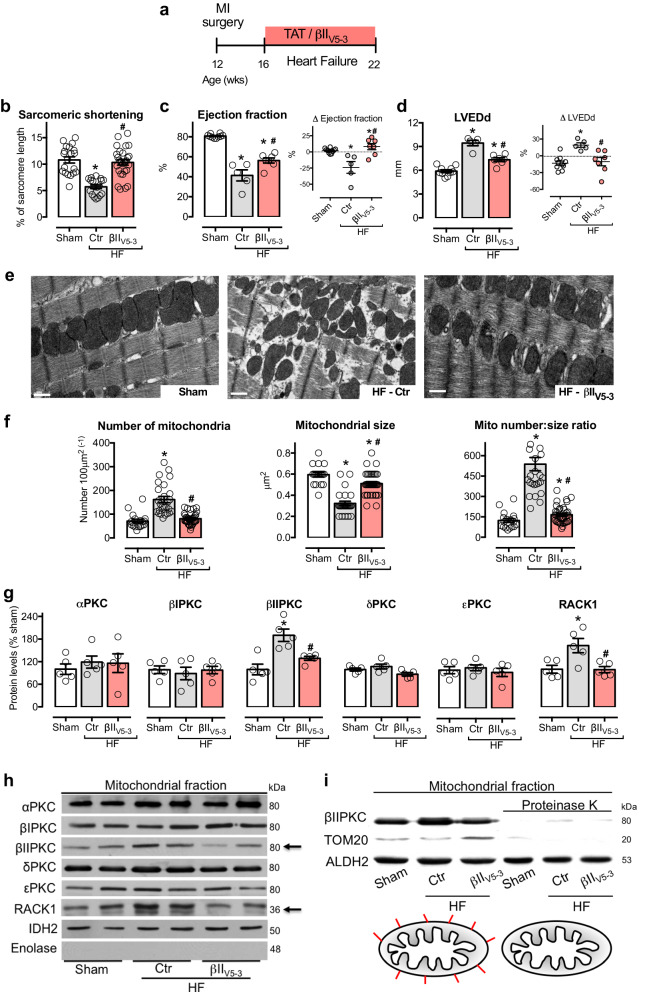


The βIPKC western blot band in Fig. 1h was duplicated and erroneously labeled as Drp1 in Fig. 3e.

The correct version of Fig. 3 is:
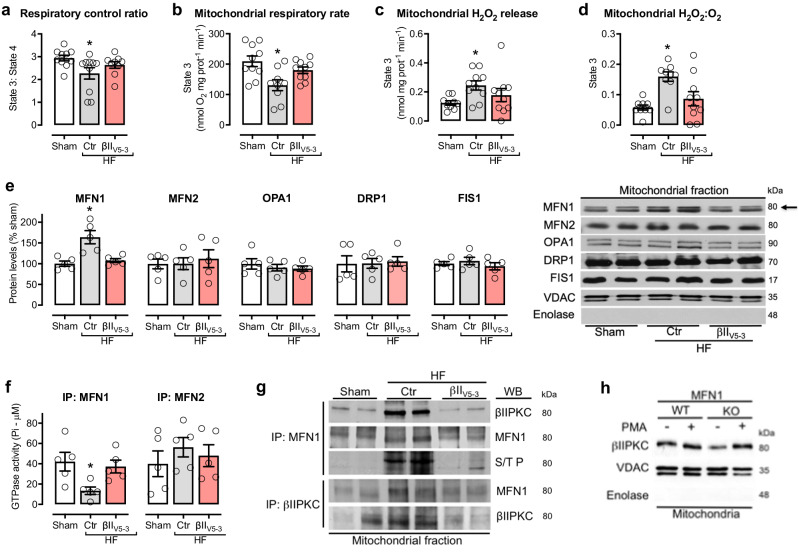


which replaces the previous incorrect version:
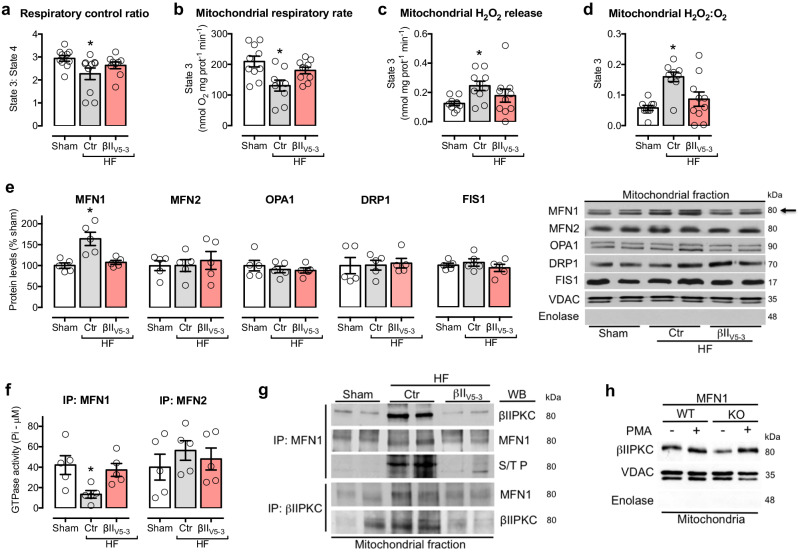


The same error occurred in the corresponding uncropped blots, where the βIPKC band in Supplementary Fig. 5a was duplicated and erroneously labeled as Drp1 in Supplementary Fig. 6.

The correct version of Supplementary Fig. 6 is:
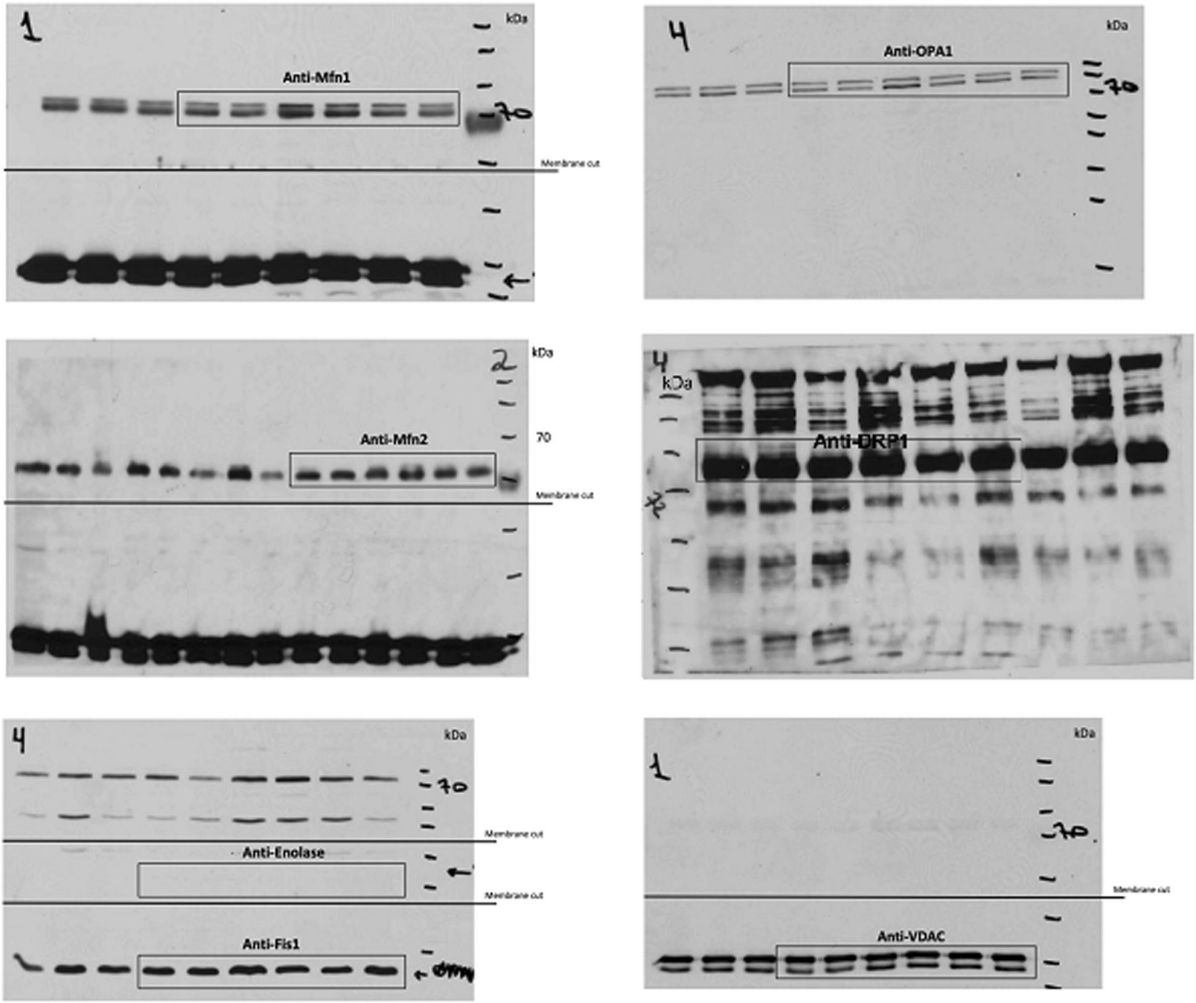


which replaces the previous incorrect version:
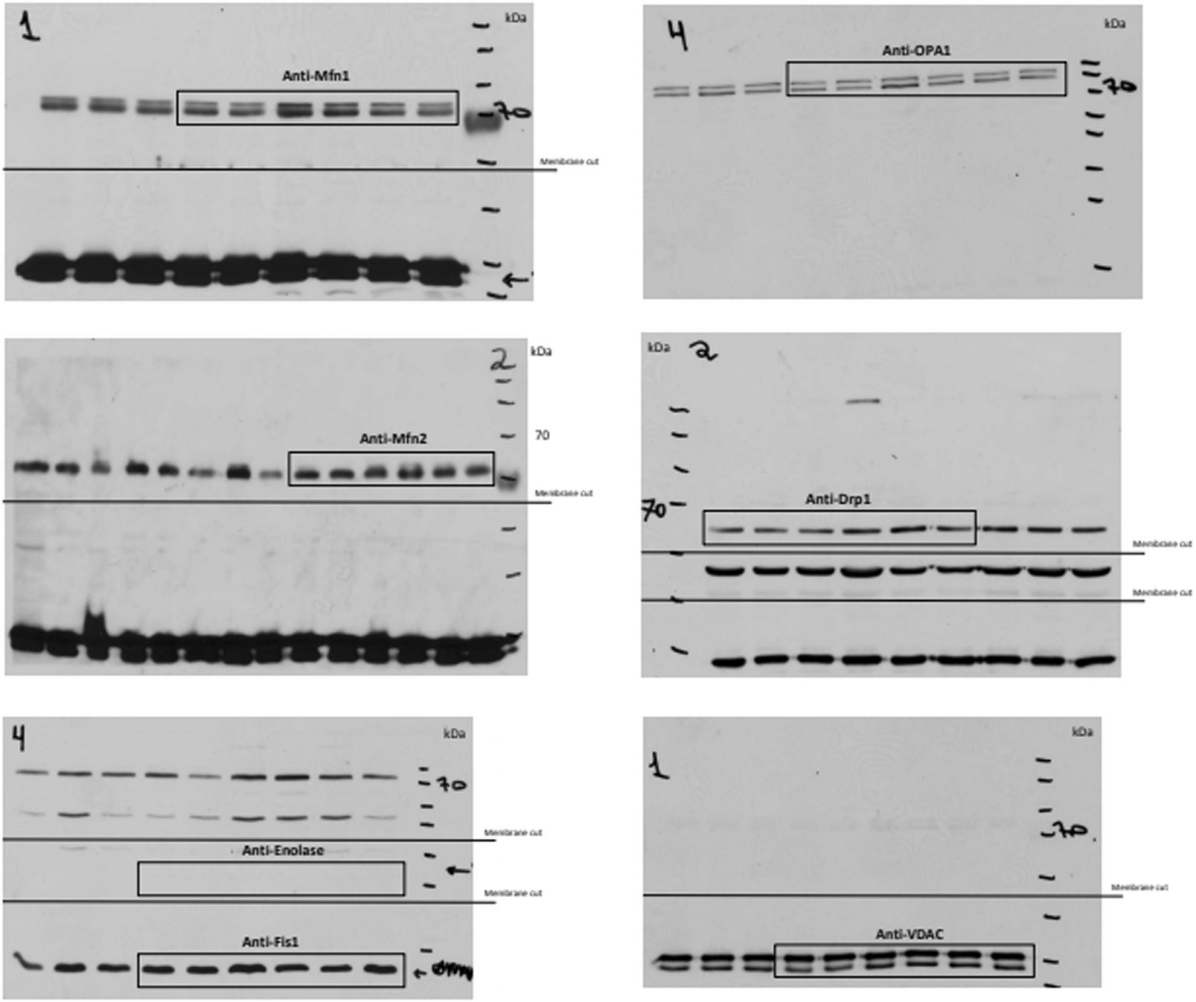


In Supplementary Fig. 8, the lanes corresponding to TOM70 have been erroneously shifted to the right and reproduced in the cropped version included in Supplementary Fig. 1.

The correct version of Supplementary Fig. 8 is:
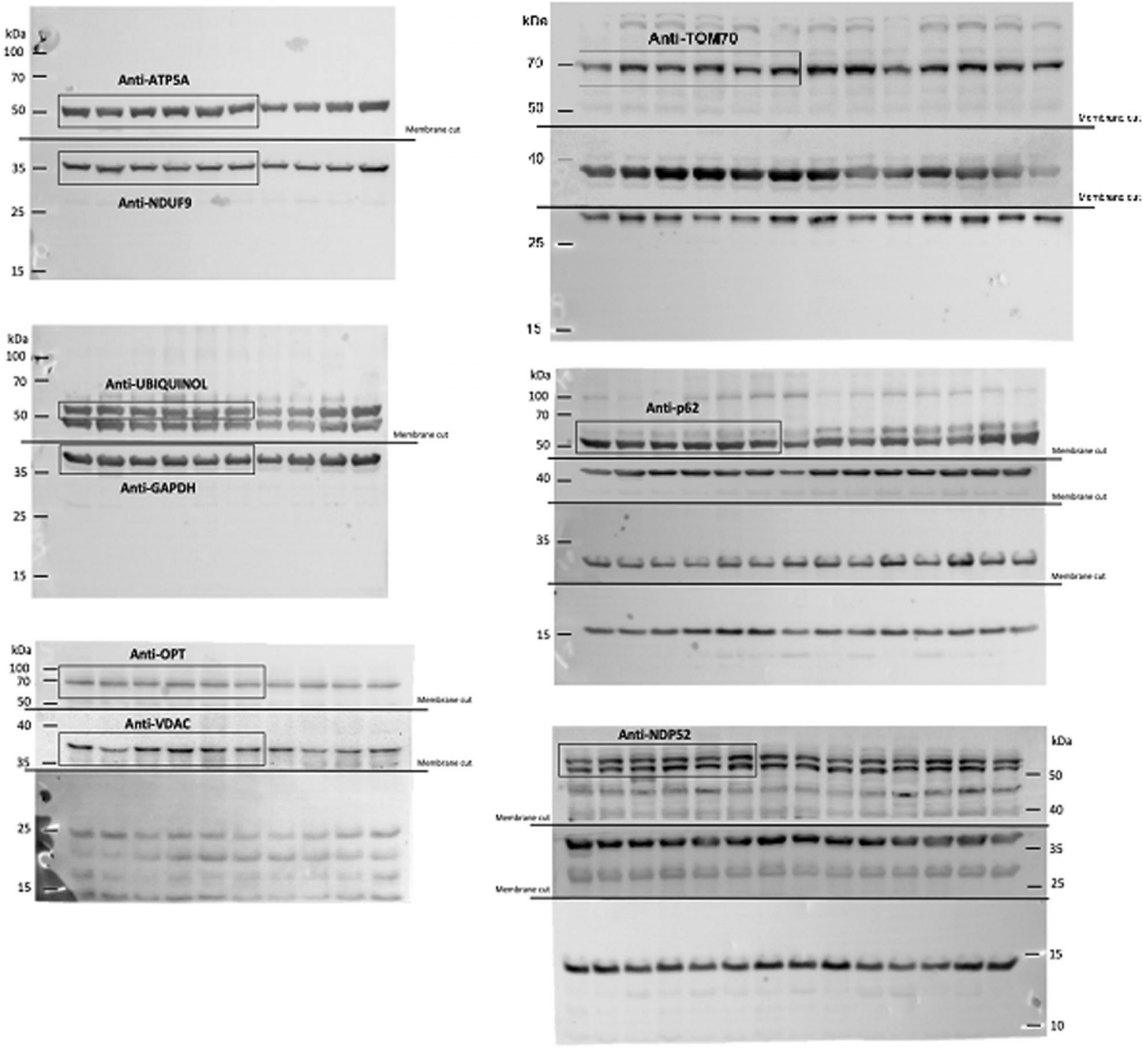


which replaces the previous incorrect version:
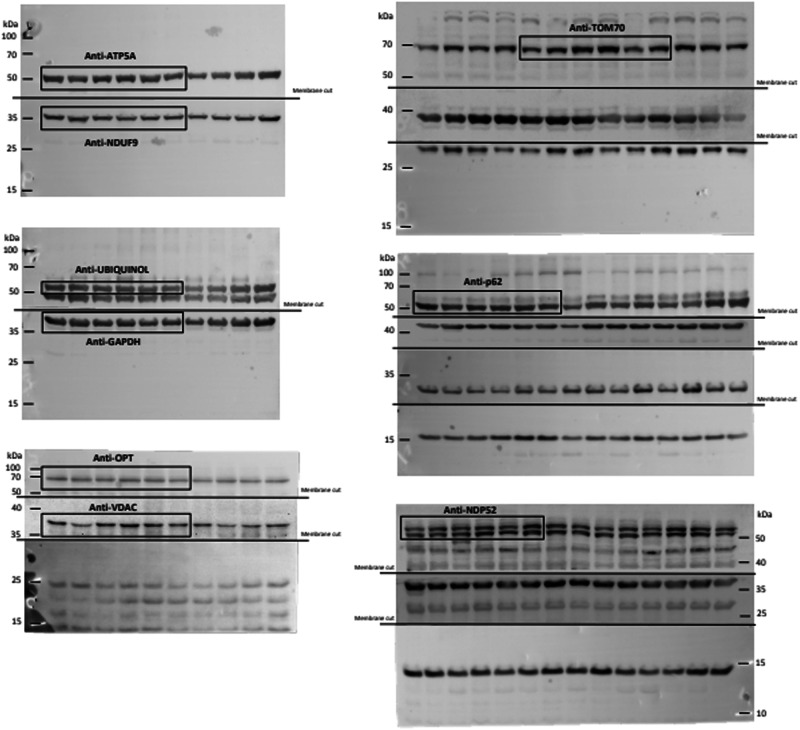


The correct version of Supplementary Fig. 1 is:
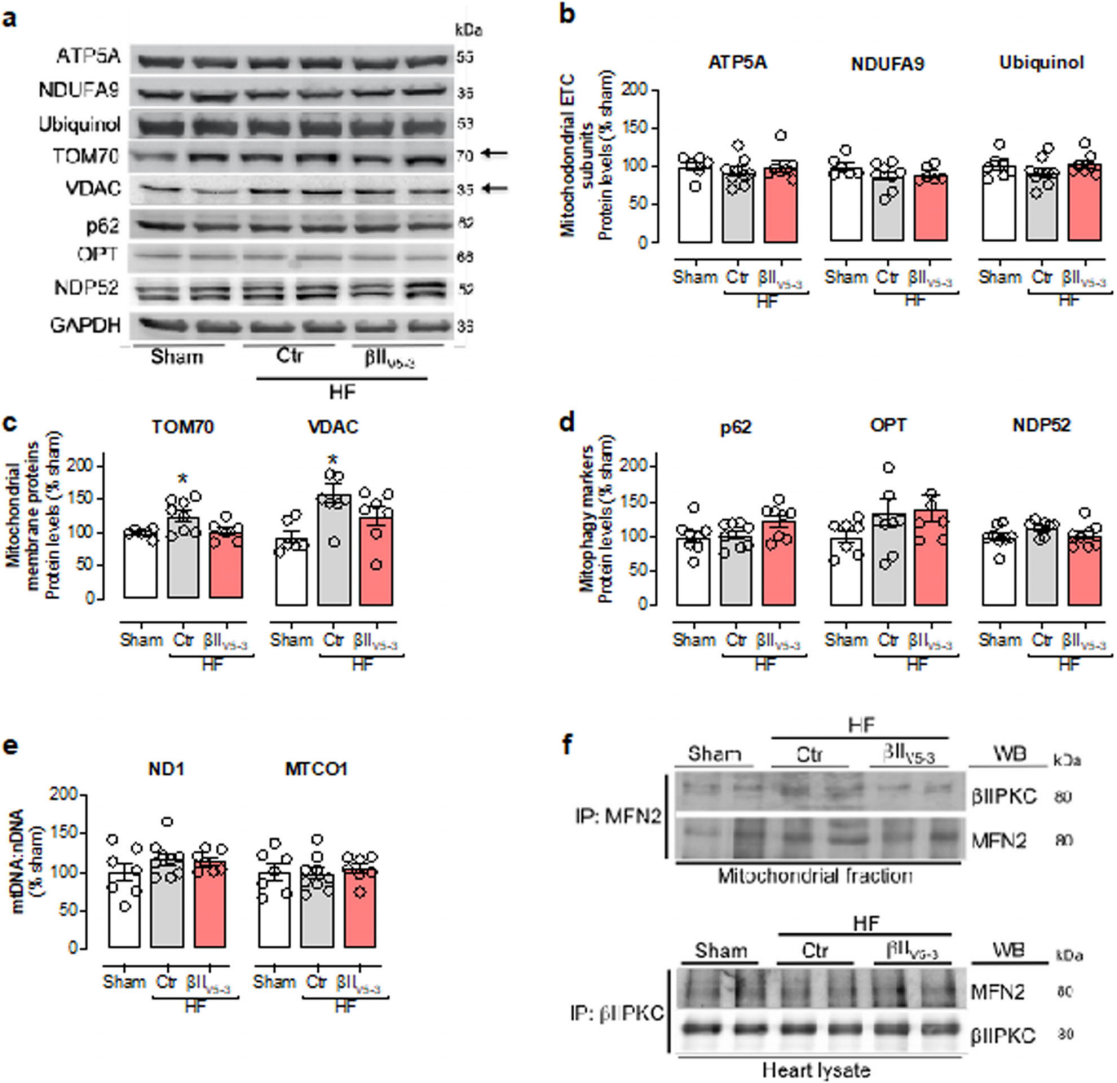


which replaces the previous incorrect version:
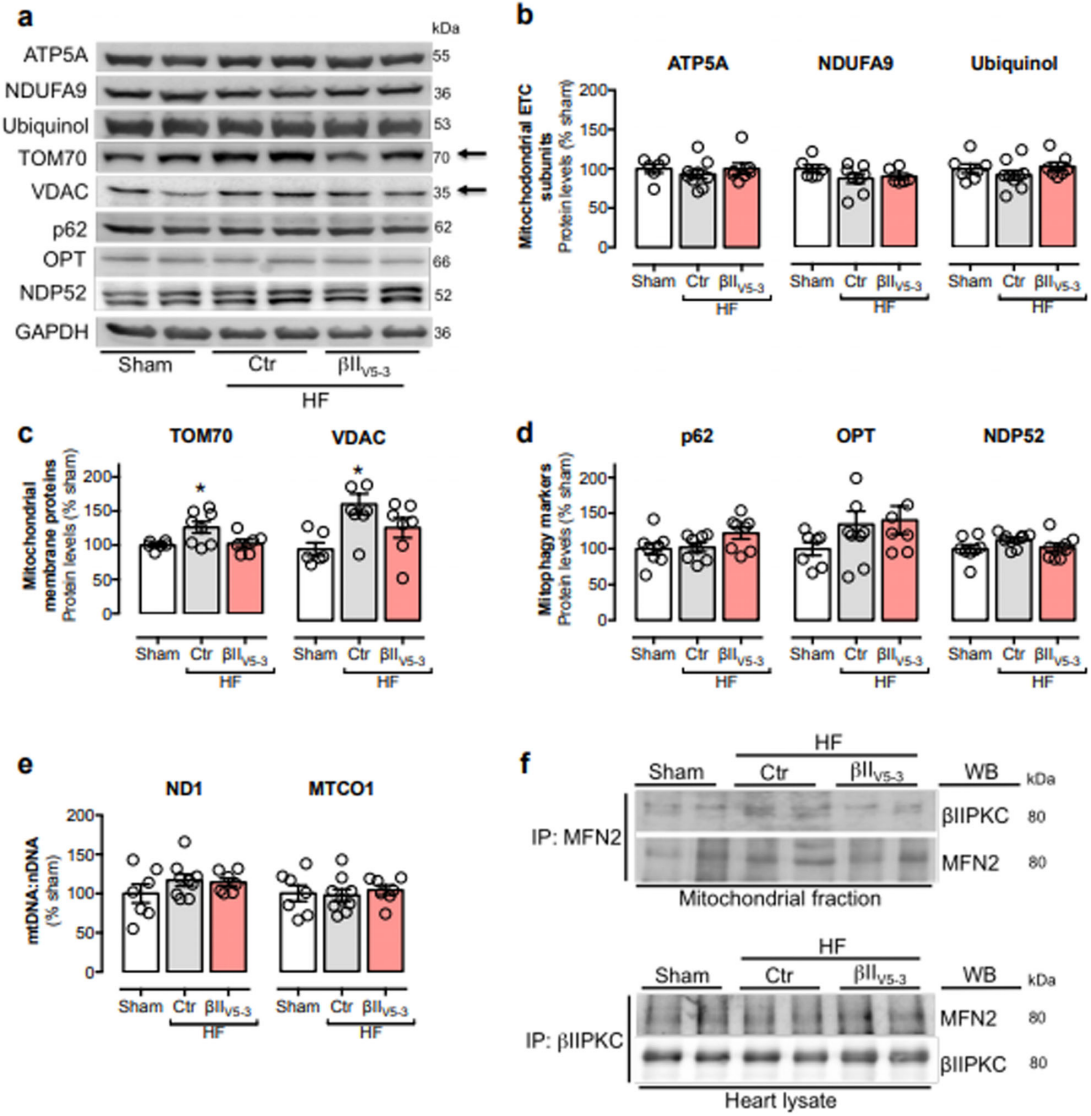


Additionally, the original version of this Article omitted the following competing interests:

‘J.C.B.F and D.M.-R. are co-inventors of patent on “Antagonists of mitofusin 1 and beta II PKC association for treating heart failure”, PCT/US2019/062854. The remaining authors declare no competing interests.’

These errors have been corrected in both the PDF and HTML versions of the Article.

Finally, the original version of this Article did not include uncropped blots corresponding to Fig. 1h (RACK1), Fig. 1i (βIIPKC, TOM20 and ALDH2) and Fig. 2d (βIIPKC, MFN1 and IDH2).

The HTML has been updated to include a corrected version of the Supplementary Information.

### Supplementary information

Updated Supplementary Information

