# Supplementary material for: A selective inhibitor of mitofusin 1-βIIPKC association improves heart failure outcome in rats
Source: Nat Commun. 2019 Jan 18;10:329. doi: 10.1038/s41467-018-08276-6 (PMC6338754; doi:10.1038/s41467-018-08276-6)
Supplement: Supplementary file 1 — Supplementary Information [file 41467_2018_8276_MOESM1_ESM.docx]

**Supplementary Information for**

**A selective inhibitor of mitofusin 1-βIIPKC association improves heart failure outcome in rats**

Ferreira *et al.*

**Supplementary Methods**

**Study design**

The study with βII_V5-3_ treatment in healthy male rats included two groups: sham, n=6; and βII_V5-3_-treated, n=6. Four weeks after sham surgery, rats were randomized to drug treatments. Each group was treated with TAT_47-57_-βII_V5-3_ peptide (global βIIPKC inhibitor, 3mg Kg^-1^ day^-1^) or equimolar concentration of TAT_47-57_-carrier peptide (negative control), using Alzet osmotic pumps under the skin on the back of the animal for sustained drug delivery, and pumps were replaced every 2 weeks. At the end of the protocol, cardiac function was re-evaluated by an observer blinded to the treatment groups. Forty-eight hours later, all the rats were euthanized by decapitation for other analyses.

**Infarct size and apoptosis**

Cardiac slices were fixed using 4% buffered formalin and embedded in paraffin for routine histological processing. 5 µm cardiac sections were stained with Masson’s trichrome and the quantification of myocardial infarct area, performed in the left ventricle free wall, was done with computer-assisted morphometric system (Leica Quantimet 500, Cambridge). The myocardial infarcted area was expressed as a percentage of the total surface area of the left ventricle.  Cell apoptosis in the myocardium was determined by TUNEL staining, according to the manufacturer's instructions (*Apoptosis Detection Kit*, Trevigem 4812/30-k). TUNEL labeling was performed in 5 µm cardiac sections, visualized using a fluorescence microscope and the data are expressed as the TUNEL-positive cardiomyocytes relative to total nuclei.

**Mitochondrial DNA**

To quantify the relative amount of mitochondrial DNA (mtDNA) per nucleus DNA (nDNA), we isolated total DNA from cardiac samples using DNeasy Blood & Tissue Kit (Qiagen 69504) according to manufacturer’s protocol. DNA was then purified by phenol-chloroform extraction and ethanol precipitation. Quantitative real time polymerase chain (qPCR) reaction were carried out separately and amplifications were performed with an ABI Prism 7500 Sequence Detection System by using Maxima® SYBR Green ROX qPCR Master Mix (Fermentas K0221). Melting point dissociation curves were used to confirm the purity of the amplification products. Results were expressed using the comparative cycle threshold (Ct) method as described by the manufacturer. Calculation of the mtDNA copy number relative do nuclear DNA (nDNA) was performed using the formula: 2 x 2ΔCt, where ΔCt is the nDNA Ct values minus mtDNA Ct values. MTCO1 and ND1 genes were used to quantify mtDNA and Rplp0 gene to nDNA. Data are expressed as the percentage of sham.

Primer sequences:

| MTCO1 | 5’ATC AAA TGA TCC CCC GCC AT3’  5’GTG GAC GAA GCC AGC TAT GA3’ |
| --- | --- |
| ND1 | 5’TGGCCTTCCTCACCCTAGTA3’  5’TTA GGG GGC GTA TGG GTT CT3’ |
| Rplp0 | 5’CCT TCC CAC TGG CTG AAA AG3’  5’GAC TTG GTG TGA GGG GCT TAG3’ |

**Statistical analysis**

Data are presented as means ± standard error of the mean (SEM). Data normality was assessed through Shapiro-Wilk test. Student t test was used to analyze data presented in Supplementary Figure 2. One-way analysis of variance (ANOVA) was used to analyze data presented in Supplementary Figure 1, Supplementary Figure 3. Two-way ANOVA for repeated measures was used to analyze data depicted in Supplementary Table 1 and Supplementary Table 3. Whenever significant F-values were obtained, Duncan adjustment was used for multiple comparison purposes. GraphPad Prism Statistics was used for the analysis and statistical significance was considered achieved when the value of P was <0.05.

**Supplementary Figures**

**Supplementary Figure 1** βIIPKC inhibition does not alter cardiac mitochondrial content in failing rat hearts. **a** Representative western blots and protein levels of **b** mitochondrial electron transport chain subunits (ATP5A, NDUF9 and Ubiquinol), **c** mitochondrial membrane proteins (TOM70 and VDAC) and **d** mitophagy markers (p62, NDP52 and OPT); **e** mitochondrial DNA copy number of MTCO1 and ND1 genes and **f** cardiac mitochondrial MFN2 immunoprecipitate probed against anti-MFN2 and βIIPKC antibodies; and βIIPKC immunoprecipitate from heart lysate probed against anti-βIIPKC and MFN2 (representative blot of three independent experiments) from heart samples of sham (white bars, n=7), TAT-treated heart failure (HF-Ctr, gray bars, n=9) and βII_V5-3_-treated heart failure (HF-βII_V5-3_, red bars, n=7). Biochemical measurements were performed in the cardiac remote (viable) zone. These measurements were performed at the end of the experimental protocol as described in Figure 1A. Data are means ± SEM. *P<0.05 *vs.* Sham. One-way analysis of variance (ANOVA) with post-hoc testing by Duncan.

**
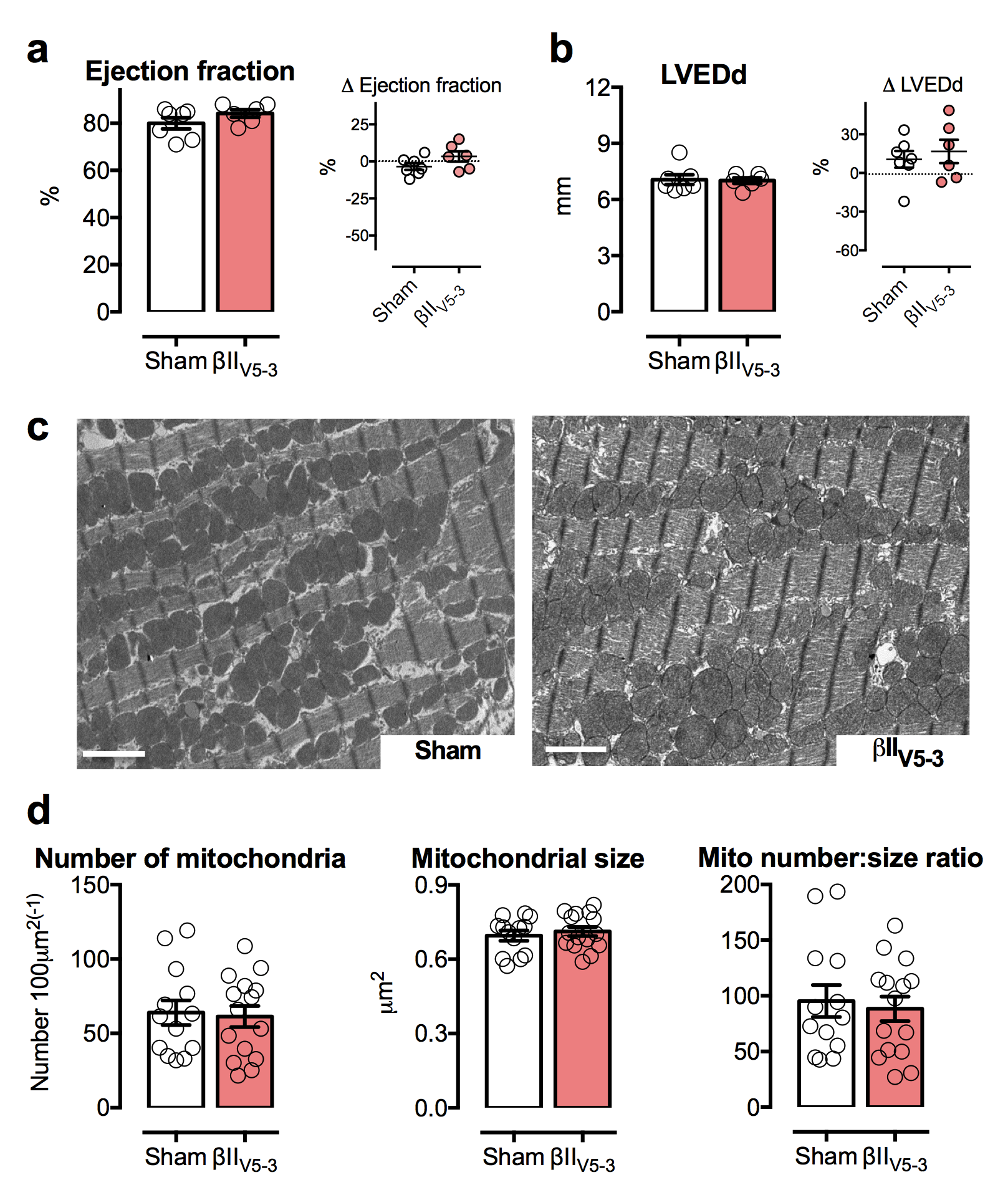
**

**Supplementary Figure 2** Sustained βIIPKC inhibition does not alter cardiac function and mitochondrial morphology in healthy (sham) rats. **a** Left ventricular ejection fraction and **b** LVEDd [left ventricular end-diastolic dimension] measured by echocardiography at the end of the experimental protocol, input: delta of measurements performed before and after treatment; **c** representative cardiac transmission electron micrographs (scale bar: 2 μm); and **d** quantification of intermyofibrillar mitochondrial number and area in the transmission electron micrographs in heart samples from sham (white bars, n=10) and βII_V5-3_-treated sham animals (βII_V5-3_, red bars, n=6). Peptide treatment was continuous (for six weeks) using an Alzet pump, delivering at a rate of 3mg Kg^-1^ day^-1^. All measurements were performed at the end of the experimental protocol as described in Figure 1A. Data are means ± SEM.

**
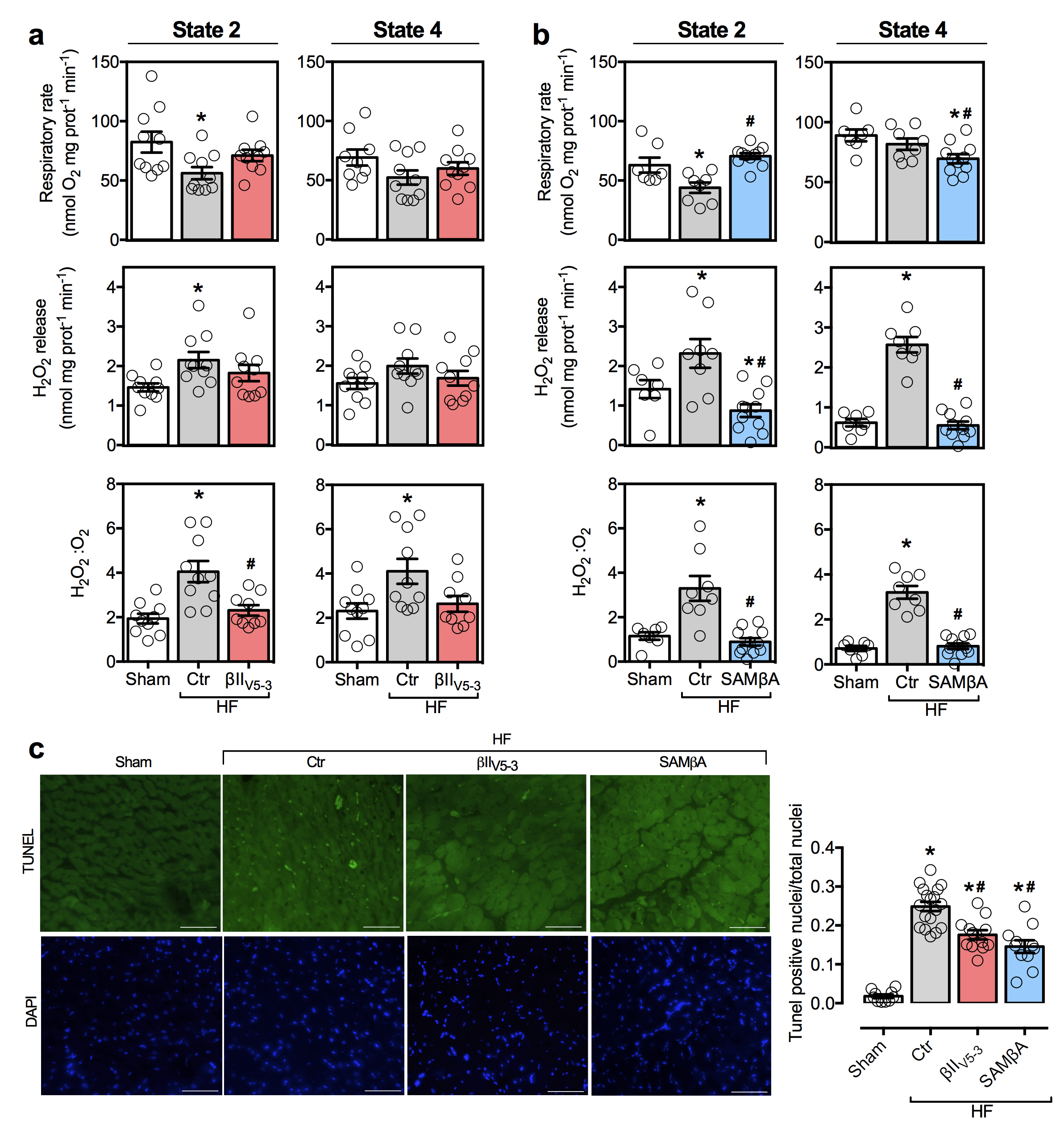
**

**Supplementary Figure 3** Inhibition of global βIIPKC (βII_V5-3_) and βIIPKC-Mfn1 protein-protein interaction (SAMβA) improves mitochondrial function in heart failure *in vivo*. **a** Mitochondrial state-dependent oxygen control rates, absolute H_2_O_2_ release and H_2_O_2:_O_2_ in heart samples from sham (white bars, n=10), TAT-treated heart failure (HF-Ctr, gray bars, n=10) and βII_V5-3_-treated heart failure (HF-βII_V5-3_, red bars, n=10). These measurements were performed at the end of the experimental protocol as described in Figure 1A. **b** Mitochondrial state-dependent oxygen control rates, absolute H_2_O_2_ release and H_2_O_2_:O_2_ in heart samples from sham (white bars, n=7), TAT-treated heart failure (HF-Ctr, gray bars, n=8) and SAMβA-treated heart failure (HF-SAMβA, blue bars, n=11). These measurements were performed at the end of the experimental protocol as described in Figure 6A. **c** Representative TUNEL-stained left ventricular sections (scale bar: 50 μm) and quantification of apoptotic cell death in heart samples from sham, HF-Ctr, HF-βII_V5-3_ and HF-SAMβA samples. Data are means ± SEM. *P<0.05 *vs.* Sham. #P<0.05 *vs.* HF-Ctr. One-way analysis of variance (ANOVA) with post-hoc testing by Duncan.

**
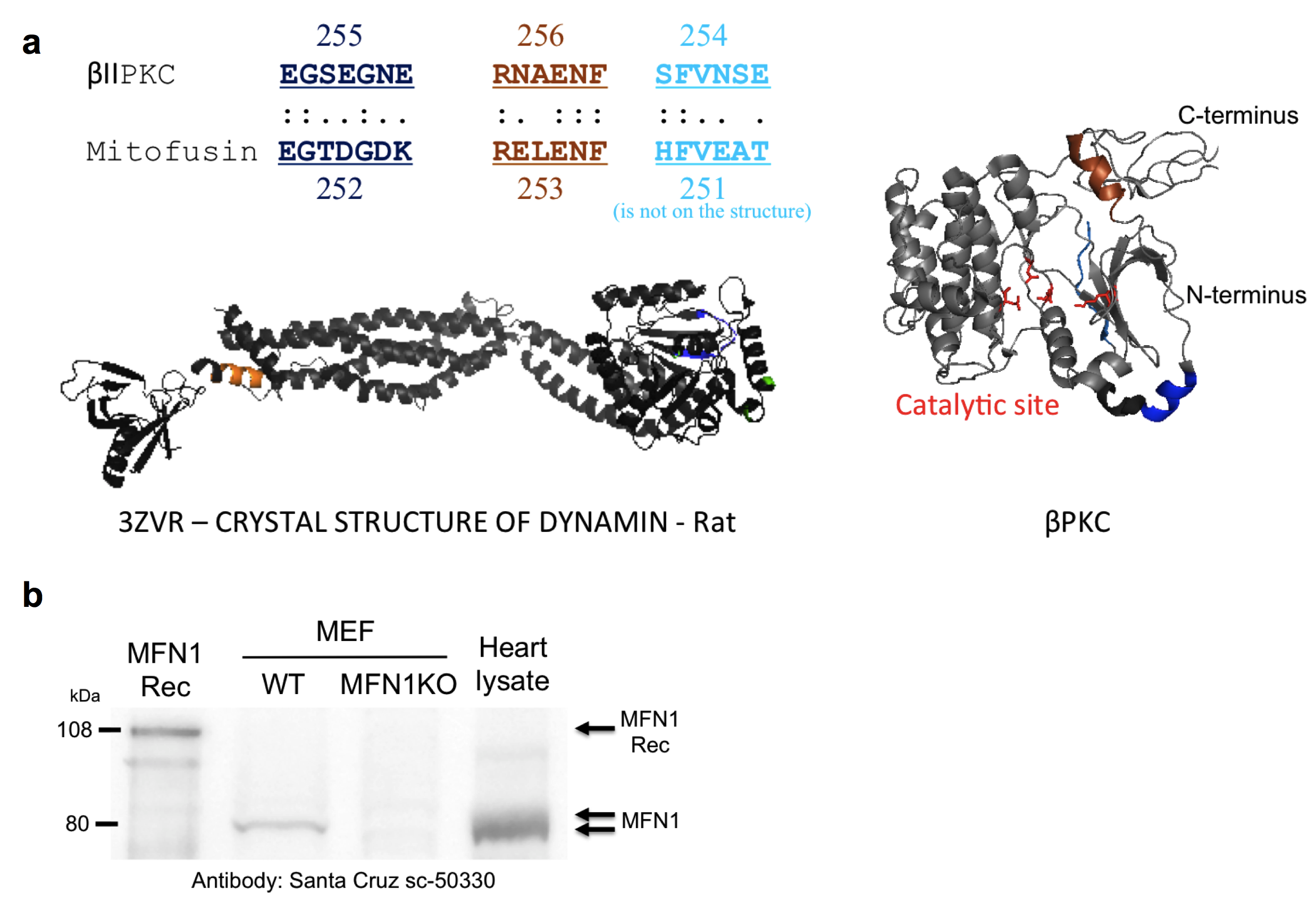
**

**Supplementary Figure 4** **a** Rational design of peptides related to protein-protein interaction derived from either βIIPKC or Mfn1. Sequence alignment of human βIIPKC and Mfn1 and the short sequences of peptides P251, P252, P253, P254, P255 and P256 (SAMβA). The colored structures indicate protein-protein interactions from each peptide, except for 251. **b** Positive (Mfn1 recombinant – Abnova cat. H00055669-P01) and negative (Mouse embryonic fibroblasts Mfn1 knockout – MFN1KO) controls for the detection of Mfn1 by western blotting. The antibody used (Santa Cruz cat. sc-50330, dilution 1:500) can recognize a double band in tissue samples.

**Supplementary Figure 5** Original source data of the blot images used in **a** Figure 1h and **b** Figure 2e.

**Supplementary Figure 6** Original source data of the blot images used in Figure 3e.

**Supplementary Figure 7** Original source data of the blot images used in **a** Figure 3h, **b** Figure 4a and **c** Figure 4e.

**Supplementary Figure 8** Original source data of the blot images used in Supplementary Figure 1a.

**Supplementary Figure 9** Original source data of the blot images used in Figure 1 h (RACK1), Figure 1i (βIIPKC, TOM20 and ALDH2) and Figure 2d (βIIPKC, MFN1 and IDH2).

**Supplementary Tables**

**Supplementary Table 1** Echocardiographic measurements before and after sustained treatment with βII_V5-3_ peptide in heart failure

|  |  | **Four weeks after MI surgery**  **(Before drug treatment begins)** | | |  | **Ten weeks after MI surgery**  **(Six weeks of treatment)** | | |
| --- | --- | --- | --- | --- | --- | --- | --- | --- |
| **Parameter** |  | **Sham** | **HF-Ctr** | **HF-βII_V5-3_** |  | **Sham** | **HF-Ctr** | **HF-βII_V5-3_** |
| **EF (%)** |  | 79.2±1.2 | 53.4±1.0* | 52.1±1.9* |  | 80.7±1.8 | 41.3±5.5*‡ | 56.3±2.5*† |
| **FS (%)** |  | 42.9±1.2 | 24.4±0.6* | 23.4±1.1* |  | 43.9±0.6 | 18.0±2.7* | 25.7±1.4*† |
| **LVEDd (mm)** |  |  | 7.94±0.29 | 7.96±0.34 |  | 5.89±0.19 | 9.44±0.34*‡ | 7.01±0.42† |
| **LVEDs (mm)** |  | 3.89±0.12 | 6.01±0.23* | 6.08±0.22* |  | 3.29±0.09 | 7.72±0.28*‡ | 5.23±0.37*† |
| **PWth (mm)** |  | 1.15±0.06 | 1.32±0.04 | 1.42±0.10 |  | 1.26±0.04 | 1.76±0.10*‡ | 1.38±0.11 |
| **IVSth (mm)** |  | 0.91±0.04 | 0.88±0.03 | 0.86±0.03 |  | 0.79±0.03 | 1.02±0.02 | 0.93±0.03 |
| **HR (bpm)** |  | 241±4 | 234±5 | 247±19 |  | 253±6 | 254±12 | 247±11 |
| **MI area (%)** |  | - | - | - |  | -  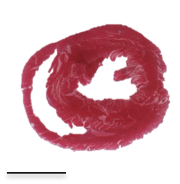 | 46.97±3.5  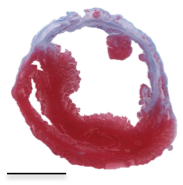 | 47.24±2.6  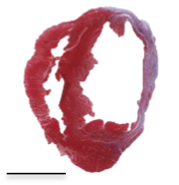 |

Left ventricular ejection fraction (EF) and fractional shortening (FS); left ventricular end-diastolic diameter (LVEDd) and end-systolic diameter (LVEDs), posterior wall thickness (PWth), interventricular septum thickness (IVSth), heart rate (HR) and myocardial infarction (MI) area and representative images (scale bar: 0.4 cm) in sham (n=10), TAT-treated heart failure (HF-Ctr, n=5) and βII_V5-3_-treated heart failure (HF-βII_V5-3_, n=7). Data are means ± SEM. *P<0.05 *vs*. Sham; †P<0.05 *vs*. HF-Ctr; ‡P<0.05 *vs*. Pre-treatment. Two-way analyses of variance (ANOVA) with post-hoc testing by Duncan. The observer was blinded to the treatment groups.

**Supplementary Table 2** Heat-map of the RNAENFDRF conservation in orthologs.

| **Parameter** |  | **Human** | **Mouse** | **Rat** | **Chicken** |
| --- | --- | --- | --- | --- | --- |
| **Protein kinase C beta II (PRKCB)** |  | RNAENFDRF | RNAENFDRF | RNAENFDRF | RNAENFDRF |
| **Protein kinase C alpha (PRKCA)** |  | KGAENFDKF | KGAENFDKF | KGAENFDKF | KGAENFDKF |
| **Protein kinase C gamma (PRKCG)** |  | RSGENFDKF | RSGENFDKF | RSGENFDKF | No Protein |
| **R3H and coiled-coil domain-containing protein 1 (R3HCC1)** |  | RTAENFDLL | RTAENFDLL | HTVNNHAFL | RTVENVDLL |
| **MAGUK p55 subfamily member 6 (VAM-1)** |  | RNAE-FDRH | RNAE-FDRH | RNAE-FDRH | No Protein |
| **Mitofusin1 (MFN1)** |  | NELENFTKQ | SELENFSKQ | RELENFSKQ | NELDHFTKH |

RNAENFDRF sequence is found in 7 proteins in the human genome. The heat-map in Figure 5G is a depiction of the data provided here, showing the conservation of RNAENFDRF in orthologs of these proteins in mouse, rat and chicken; RNAENFDRF is conserved only in βIIPKC. The following amino acid substitutions were consider conserved for the analyses: G↔P; Y↔F; I↔L↔V; M↔C; S↔T; N↔Q; D↔E and R↔K.

**Supplementary Table 3** Echocardiographic measurements before and after sustained treatment with SAMβA peptide in heart failure

|  |  | **Four weeks after MI surgery**  **(Before drug treatment begins)** | | |  | **Ten weeks after MI surgery**  **(Six weeks of treatment)** | | |
| --- | --- | --- | --- | --- | --- | --- | --- | --- |
| **Parameter** |  | **Sham** | **HF-Ctr** | **HF-SAMβA** |  | **Sham** | **HF-Ctr** | **HF-SAMβA** |
| **EF (%)** |  | 82.6±0.5 | 49.7±2.4* | 51.4±2.1* |  | 82.1±1.1 | 37.9±3.3*‡ | 62.3±3.1*† |
| **FS (%)** |  | 45.9±0.5 | 22.2±1.2* | 23.1±1.1* |  | 45.5±1.2 | 16.3±1.5* | 30.0±2.0*† |
| **LVEDd (mm)** |  | 5.58±0.27 | 8.91±0.19* | 8.60±0.46* |  | 5.86±0.29 | 10.46±0.24* | 7.67±0.43*† |
| **LVEDs (mm)** |  | 3.03±0.16 | 6.94±0.23* | 6.65±0.42* |  | 3.19±0.18 | 8.75±0.23*‡ | 5.40±0.40*† |
| **PWth (mm)** |  | 1.27±0.04 | 1.42±0.08 | 1.38±0.08 |  | 1.32±0.07 | 1.79±0.09*‡ | 1.4 8±0.06 |
| **IVSth (mm)** |  | 0.93±0.03 | 0.97±0.03 | 0.99±0.09 |  | 0.91±0.05 | 0.94±0.03 | 0.96±0.03 |
| **HR (bpm)** |  | 247±8 | 278±17 | 263±10 |  | 262±14 | 280±8 | 269±12 |
| **SBP (mmHg)** |  | 127±4.7 | 119±5.4 | 119±4.9 |  | 123±3.3 | 117±2.4 | 117±2.5 |
| **MI area (%)** |  | - | - | - |  | -  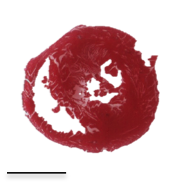 | 46.99±3.77  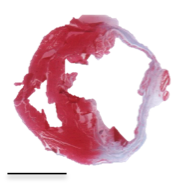 | 46.03±5.27  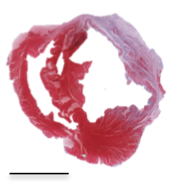 |

Left ventricular ejection fraction (EF) and fractional shortening (FS); left ventricular end-diastolic diameter (LVEDd) and end-systolic diameter (LVEDs), posterior wall thickness (PWth), interventricular septum thickness (IVSth), heart rate (HR), systolic blood pressure (SBP) and myocardial infarction (MI) area and representative images (scale bar: 0.4 cm) in sham (n=7), TAT-treated heart failure (HF-Ctr, n=8) and SAMβA-treated heart failure (HF-SAMβA, n=11). Data are means ± SEM. *P<0.05 *vs*. Sham; †P<0.05 *vs*. HF-Ctr; ‡P<0.05 *vs*. Pre-treatment. Two-way analyses of variance (ANOVA) with post-hoc testing by Duncan. The observer was blinded to the treatment groups.
