# Supplementary material for: A selective inhibitor of mitofusin 1-βIIPKC association improves heart failure outcome in rats
Source: Nat Commun. 2019 Jan 18;10:329. doi: 10.1038/s41467-018-08276-6 (PMC6338754; doi:10.1038/s41467-018-08276-6)
Supplement: Supplementary file 3 — Reporting Summary [file 41467_2018_8276_MOESM3_ESM.pdf]

nature research

Corresponding author(s): Julio Cesar Batista Ferreira, Daria Mochly-Rosen

Reporting Summary

Nature Research wishes to improve the reproducibility of the work that we publish. This form provides structure for consistency and transparency in reporting. For further information on Nature Research policies, see Authors & Referees and the Editorial Policy Checklist.

Statistical parameters

When statistical analyses are reported, confirm that the following items are present in the relevant location (e.g. figure legend, table legend, main text, or Methods section).

n/a	Confirmed	
☐	☒	The exact sample size (*n*) for each experimental group/condition, given as a discrete number and unit of measurement
☐	☒	An indication of whether measurements were taken from distinct samples or whether the same sample was measured repeatedly
☐	☒	The statistical test(s) used AND whether they are one- or two-sided<br>*Only common tests should be described solely by name; describe more complex techniques in the Methods section.*
☐	☒	A description of all covariates tested
☐	☒	A description of any assumptions or corrections, such as tests of normality and adjustment for multiple comparisons
☐	☒	A full description of the statistics including central tendency (e.g. means) or other basic estimates (e.g. regression coefficient) AND variation (e.g. standard deviation) or associated estimates of uncertainty (e.g. confidence intervals)
☐	☒	For null hypothesis testing, the test statistic (e.g. *F*, *t*, *r*) with confidence intervals, effect sizes, degrees of freedom and *P* value noted<br>*Give P values as exact values whenever suitable.*
☒	☐	For Bayesian analysis, information on the choice of priors and Markov chain Monte Carlo settings
☒	☐	For hierarchical and complex designs, identification of the appropriate level for tests and full reporting of outcomes
☒	☐	Estimates of effect sizes (e.g. Cohen's *d*, Pearson's *r*), indicating how they were calculated
☐	☒	Clearly defined error bars<br>*State explicitly what error bars represent (e.g. SD, SE, CI)*

Our web collection on statistics for biologists may be useful.

Software and code

Policy information about availability of computer code

Data collection	Prism Graphad software version 6.0 or Microsoft Excel (v. 16) was used to collect the data.
Data analysis	Prism Graphad software version 6.0 was used to analyze the data. No custom software or code was used.

For manuscripts utilizing custom algorithms or software that are central to the research but not yet described in published literature, software must be made available to editors/reviewers upon request. We strongly encourage code deposition in a community repository (e.g. GitHub). See the Nature Research guidelines for submitting code & software for further information.

Data

Policy information about availability of data

All manuscripts must include a data availability statement. This statement should provide the following information, where applicable:

- Accession codes, unique identifiers, or web links for publicly available datasets
- A list of figures that have associated raw data
- A description of any restrictions on data availability

The statement is provided in the Method section.

April 2018

Field-specific reporting

Please select the best fit for your research. If you are not sure, read the appropriate sections before making your selection.

☒ Life sciences ☐ Behavioural & social sciences ☐ Ecological, evolutionary & environmental sciences

For a reference copy of the document with all sections, see nature.com/authors/policies/ReportingSummary-flat.pdf

Life sciences study design

All studies must disclose on these points even when the disclosure is negative.

Sample size	No sample-size calculation was performed.
Data exclusions	For animal studies, no animals were excluded from the study. For in vitro and cell-based assays, no data were excluded from the experiments and analysis.
Replication	Most experiments in in vitro and cell-based assays were repeated in at least three independent experiments and the data were reproducible.
Randomization	For cell-based assays, cells were plated and distributed at equal density for treatment and control groups. The confluence of the cells at the time of treatment was noted to be equal and the allocation of treatment was randomly assigned.<br>For animal studies, Sham and myocardial infarction surgeries were done on the same day, usually 10 rats per day. Four weeks after myocardial infarction, rats were randomized to drug treatments.
Blinding	For all cardiac function analysis in rats, the observer was blinded to treatment conditions.

Reporting for specific materials, systems and methods

Materials & experimental systems

n/a	Involved in the study	
☒	☐	Unique biological materials
☐	☒	Antibodies
☐	☒	Eukaryotic cell lines
☒	☐	Palaeontology
☐	☒	Animals and other organisms
☒	☐	Human research participants

Methods

n/a	Involved in the study	
☒	☐	ChIP-seq
☒	☐	Flow cytometry
☒	☐	MRI-based neuroimaging

Antibodies

Antibodies used

Drp1 (clone), BD Biosciences, 611113, LOT 25726
GAPDH (6C5), Advanced Immunochemical, 6c5, LOT 13/06-III-G4C5
Mfn1 (3C9), Abnova, H00055669-M04, LOT 11284-3C9
Mfn2 (6A8), Abnova, H00009927-M01, LOT 11340-4H8
Opa1 (8A8) Abnova, H00004976-M01, LOT 10145-8A8
IDH2 (5F11), Abnova, H00003418-M01, LOT 11104-5F11
ATP5A (15H4C4), Abcam, Ab14748, LOT GR 209582-7
NDUFA9 (20C11B11B11), Abcam, Ab14713, LOT GR 200432-4
Ubiquinol (16D10AD9AH5), Abcam, Ab110252, LOT GR 47101-2
Troponin I, Abcam, Ab47003
SQSTM1/p62, Cell Signaling, 5114, LOT 4
Phospho-Troponin I (serine 23/24), Cell Signaling, 4004, LOT
Optineurin, Proteintech, 10837-1-AP, LOT 00049904
NDP52-CALCOCO2, Proteintech, 12229-1-AP, LOT 00040932
Fis1, Enzo Life Sciences, 210-907-R100, LOT L26384
αPKC (C-20), Santa Cruz biotechnology, sc-208, LOT L0811
βIPKC (C-16), Santa Cruz biotechnology, sc-209, LOT K1711
βIIPKC (C-18), Santa Cruz biotechnology, sc-210, LOT D2911
δPKC (C-17), Santa Cruz biotechnology, sc-213, LOT C2411
εPKC (C-15), Santa Cruz biotechnology, sc-214
RACK1 (H-187), Santa Cruz biotechnology, sc-10775

	Tom 20 (C-20), Santa Cruz biotechnology, sc-11415, LOT A3113<br>VDAC1 (D-16), Santa Cruz biotechnology, sc-32063, LOT L1212<br>Enolase (H-300), Santa Cruz biotechnology, SC-15343 LOT C1915<br>ALDH2 (3D12), Santa Cruz biotechnology, sc-1000496<br>Mfn1 (65), sc-50330, Santa Cruz biotechnology, LOT E1011
Validation	Drp1 (clone), BD Biosciences, 611113, LOT 25726 - Recommended for detection of Drp1 of mouse, rat, dog and human origin by WB and IF (citation: PMID: 20463395, PMID: 17003040, PMID: 17332775, PMID: 15979461, PMID: 17606867, PMID: 9472031).<br>GAPDH (6C5), Advanced Immunochemical, 6c5, LOT 13/06-III-G4C5 - Recommended for detection of GAPDH of mouse, rabbit, cat, rat, fish and human origin by WB, IHC and ELISA.<br>Mfn1 (3C9), Abnova, H00055669-M04, LOT 11284-3C9 - Recommended for detection of Mfn1 of human origin by WB and ELISA (citation: PMID: 28712724, PMID: 27050458, PMID: 28340937, PMID: 25336644, PMID: 24513856).<br>Mfn2 (6A8), Abnova, H00009927-M01, LOT 11340-4H8 - Recommended for detection of Mfn2 of human, mouse and rat origin by WB, IHC-P and ELISA (citation: PMID: 29464885, PMID: 29212658, PMID: 28698145, PMID: 25347680).<br>Opa1 (8A8) Abnova, H00004976-M01, LOT 10145-8A8 - Recommended for detection of Opa1 of mouse, rat and human origin by WB origin by WB and ELISA.<br>IDH2 (5F11), Abnova, H00003418-M01, LOT 11104-5F11 - Recommended for detection of IDH2 of human origin by WB, IHC-P and ELISA (citation: PMID: 26384468, PMID: 24164308).<br>ATP5A (15H4C4), Abcam, Ab14748, LOT GR 209582-7 - Recommended for detection of ATP5A of mouse, rat, cow, human, pig, caenorhabditis elegans, drosophila melanogaster and monkey origin by WB, ICC, IP, IHC-P, ICC/IF, Flow Cyt (citation: PMID: 27529784, PMID: 26824698).<br>NDUFA9 (20C11B11B11), Abcam, Ab14713, LOT GR 200432-4 - Recommended for detection of NDUFA9 of mouse, rat, cow, and human origin by WB, IHC-P, Flow Cyt (citation: PMID:28671271, PMID: 29780003).<br>Ubiquinol (16D10AD9AH5), Abcam, Ab110252, LOT GR 47101-2- Recommended for detection of Ubiquinol-Cytochrome C Reductase Core Protein I of mouse, rat, cow, and human origin by WB, ICC/IF, Flow Cyt (citation: PMID: 29222160, PMID: 29949756).<br>Troponin I, Abcam, Ab47003 - Recommended for detection of cardiac Troponin I of mouse, rat, human and pig origin by WB, IHC-P, ICC/IF, ELISA, Flow Cyt (citation: PMID: 29552383, PMID: 29593308).<br>SQSTM1/p62, Cell Signaling, 5114, LOT 4 - Recommended for detection of p62 of mouse, rat, human and monkey origin by WB (citation: PMID: 30364204, PMID: 30271755).<br>Phospho-Troponin I (serine 23/24), Cell Signaling, 4004 - Recommended for detection of Phospho-Troponin I of mouse, rat and human origin by WB (citation: PMID: 28469574, PMID: 28796250).<br>Optineurin, Proteintech, 10837-1-AP, LOT 00049904 - Recommended for detection of optineurin of mouse, rat and human origin by WB, IHC, IP, IF (citation: PMID: 26266977, PMID: 28294115, PMID: 26919428).<br>NDP52-CALCOCO2, Proteintech, 12229-1-AP, LOT 00040932 - Recommended for detection of CALCOCO2 of mouse, rat and human origin by WB, IHC, IP, IF (citation: PMID: 26919428, PMID: 26350966, PMID: 28965816).<br>Fis1, Enzo Life Sciences, 210-907-R100, LOT L26384 - Recommended for detection of Fis1 of mouse, rat and human origin by WB (citation: PMID: 28826719, PMID: 26014431, PMID: 25836420).<br>αPKC (C-20), Santa Cruz biotechnology, sc-208, LOT L0811 - Recommended for detection of αPKC of mouse, rat and human origin by WB, IP, IF, ELISA (citation: PMID: 26926225, PMID: 26398746 , PMID: 25761241).<br>βIPKC (C-16), Santa Cruz biotechnology, sc-209, LOT K1711 - Recommended for detection of βIPKC of mouse, rat and human origin by WB, IP, IF, ELISA (citation: PMID: 25077539, PMID: 25292213).<br>βIIPKC (C-18), Santa Cruz biotechnology, sc-210, LOT D2911 - Recommended for detection of βIIPKC of mouse, rat and human origin by WB, IP, IF, IHC-P, ELISA (citation: PMID: 26639163 , PMID: 23778835, PMID: 22730532).<br>δPKC (C-17), Santa Cruz biotechnology, sc-213, LOT C2411 - Recommended for detection of δIIPKC of mouse, rat and human origin by WB, IP, IF, IHC-P, ELISA (citation: PMID:24828530, PMID: 26769967, PMID: 23845726).<br>εPKC (C-15), Santa Cruz biotechnology, sc-214 - Recommended for detection of εIIPKC of mouse, rat, human and avian origin by WB, IP, IF, IHC-P, ELISA (citation: PMID: 26769967, PMID: 25778903, PMID: 29439667).<br>RACK1 (H-187), Santa Cruz biotechnology, sc-10775 - Recommended for detection of RACK1 of mouse, rat, human and avian origin by WB, IP, IF, IHC-P (citation: PMID: 22903704, PMID: 19158384, PMID: 16849317).<br>Tom 20 (C-20), Santa Cruz biotechnology, sc-11415, LOT A3113 - Recommended for detection of Tom 20 of mouse, rat, human and avian origin by WB, IP, IF, IHC-P (citation: PMID: 27390127, PMID: 26119781, PMID: 25997101).<br>VDAC1 (D-16), Santa Cruz biotechnology, sc-32063, LOT L1212 - Recommended for detection of VDAC1 of mouse, rat, human and avian origin by WB, IP, IF, IHC-P, ELISA (citation: PMID: 23376484, PMID: 21470976, PMID: 18068667).<br>Enolase (H-300), Santa Cruz biotechnology, SC-15343, LOT C1915 - Recommended for detection of enolase of mouse, rat and human origin by WB, IP, IF and IHC (citation: PMID 26268247, PMID 26025878, PMID 25053255, PMID 23589302, PMID 22272336, PMID 22354986, PMID 21779095, PMID 21307348, PMID 23885189, PMID 20217863).<br>ALDH2 (3D12), Santa Cruz biotechnology, sc-1000496 - Recommended for detection of ALDH2 of mouse, rat and human origin by WB, IP, ELISA (citation: PMID: 30015964, PMID: 25406860, PMID: 28962150).<br>Mfn1 (H65), sc-50330, Santa Cruz biotechnology, LOT E1011 - Recommended for detection of Mfn1 of mouse, rat and human origin by WB, IP, IF, IHC-P, ELISA (citation: PMID: 26538029, PMID: 25060553, PMID: 25147362).

Eukaryotic cell lines

Policy information about cell lines

Cell line source(s)	MEF (mouse embryonic fibroblasts). WT (ATCC CRL-2991) and Mfn1 knockout (ATCC CRL-2992) MEFs were acquired from ATCC and cultured in Dulbecco's Modification of Eagle's Medium/Ham's F-12 50/50 Mix supplemented with 10% FBS, 100 U mL-1 penicillin, and 100 ug mL-1 streptomycin.
Authentication	None of the cell lines used were authenticated.
Mycoplasma contamination	Cell lines were not tested for mycoplasma contamination.

Commonly misidentified lines (See ICLAC register)	We did not use any commonly misidentified cell lines.

Animals and other organisms

Policy information about studies involving animals; ARRIVE guidelines recommended for reporting animal research

Laboratory animals	Male Wistar Rats (12-22 weeks old) were maintained in a 12:12 h light-dark cycle and temperature-controlled environment (22 ° C) with free access to standard laboratory chow and tap water.
Wild animals	The study did not involve wild animals.
Field-collected samples	The study did not involve field-collected samples.
